# Hill’s Compound for Filling Teeth

**Published:** 1849-07

**Authors:** 


### HILL'S COMPOUND FOR FILLING TEETH.

We acknowledge the receipt of a specimen of this article, and intended ere this, having tested its properties; but our patients so generally prefer gold, that we have had but little opportunity. We however recently met with a tooth filled with the same article about a year since. It was the first permanent molar below, for a Miss, perhaps ten years of age; decay in the center quite large. The tooth, she said, had been tender, and presented the appearance at first view, as having been plugged with amalgam or tin; but we found the surface of the plug rather softer than tin; and as it was a tooth which, on a careful examination of the mouth we thought best to preserve, the plug was removed. The cavity had evidently not been well cleaned out, yet the composition had excluded the moisture, and answered very well the purpose of a temporary filling, so that we should not hesitate, under some circumstances, to use the article for the filling of deciduous teeth.—[CIN. ED.

